# Supplementary material for: Spatially resolved photoluminescence analysis of the role of Se in CdSexTe1−x thin films
Source: Nat Commun. 2024 Oct 9;15:8729. doi: 10.1038/s41467-024-52889-z (PMC11461497; doi:10.1038/s41467-024-52889-z)
Supplement: Supplementary file 1 — Supplementary Information [file 41467_2024_52889_MOESM1_ESM.pdf]

# Supplementary Information: Spatially resolved photoluminescence analysis of the role of Se in $CdSe_xTe_{1-x}$ thin films

**A. R. Bowman[1,2,3*], J. F. Leaver[4*], K. Frohna[2,3], S. D. Stranks[2,3], G. Tagliabue[1+] and J. D. Major[4+]**

1. Laboratory of Nanoscience for Energy Technologies (LNET), STI, École Polytechnique Fédérale de Lausanne (EPFL), Lausanne 1015, Switzerland
2. Cavendish Laboratory, Department of Physics, University of Cambridge, J.J. Thomson Avenue, Cambridge, CB3 0HE, UK
3. Department of Chemical Engineering & Biotechnology, University of Cambridge, Philippa Fawcett Drive, Cambridge, CB3 0AS, UK
4. Stephenson Institute for Renewable Energy, University of Liverpool, Liverpool, L69 7ZF, UK

*These authors contributed equally to this work

+corresponding authors: giulia.tagliabue@epfl.ch & jonmajor@liverpool.ac.uk

*Supplementary Information Note 1 – luminescence with charge extraction layers present*

We study samples on glass to remove charge extraction effects from our spectroscopic results. Here we briefly discuss additional effects that can be observed with charge extraction layers or at interfaces between two electronically active materials, noting that this is an area of ongoing spectroscopic research. Specifically, we give two examples from complementary fields.

Our first example is time resolved photoluminescence (TRPL) from halide perovskites. Several studies have focused on changes to TRPL signals when a halide perovskite is on glass compared to on a charge extraction layer, including Stolterfoht *et al*[1]. Here it was observed that a halide perovskite had significantly shorter TRPL lifetime when placed on a charge extraction layer (see figure 3 in this paper), noting that shorter TRPL lifetimes are equivalent to weaker luminescence signals in steady state luminescence. The authors attribute this reduced lifetime to a mixture of charge extraction and interfacial recombination. Importantly, while TRPL lifetimes are significantly longer following interfacial passivation (see figure 5c and 5f in this paper), there is still a rapid initial drop and charge lifetime is still lower than on glass. This shows that a reduction in TRPL lifetime (i.e. luminescence) can be due to charge extraction or interfacial charge traps, and it is difficult to deconvolute these competing effects. This has been further explored by a number of others in this field[2,3], generally demonstrating that charge transport layers can have a multitude of effects on the strength of luminescence signals. Notably, significant changes are seen even with a single transport layer deposited on the active layer, which is a form of open circuit (likely due to interfacial recombination and some charges being extracted to the transport layer and then subsequently undergoing non-radiative recombination across the interface).

Our second example is from organic semiconductors. Here it is common to study heterojunctions between two electronically active materials. Below-bandgap photoluminescence peaks have been observed due to charge transfer states between two organic semiconductors, for example as shown by Ng *et al*[4] . This demonstrates that when multiple electronically active materials are present their interaction can introduce interfacial luminescence states. By studying bare CdTe samples on glass we are able to rule out that the below-bandgap luminescence states seen in our system originate from a charge transfer state or similar.

Both these examples demonstrate the importance of studying samples on glass (or other inert substrate) when focusing on the effect of a single material within a solar cell device stack.

*Supplementary Information Note 2 – morphological characterisation*

## 1. *Secondary Ion Mass spectrometry (SIMS)*

SIMS was used to record the vertical distribution of Se and Te throughout films. Specifically, we studied Cl treated samples fabricated with 50 nm CdSe and 200 nm CdSe underlayers (noting the time intensive nature of this measurement prevented us from also exploring 100 nm CdSe samples). We recorded the intensity of Se and Te present in the film, then used a Cs+ ion beam to remove a thin layer of the film repeatedly (see methods), building a vertical profile. This process was repeated until we reached the substrate, following previous analyses of $CdSe_xTe_{1-x}$ samples[5]. We present our results in Supplementary Figure 1, for three regions measured in the 50 nm CdSe sample and two regions measured in the 200 nm CdSe sample, as a function of the etch number in the measurement. The absolute intensities of the Se and Te signals were found to vary between samples, as is presented in Supplementary Figure 1a and 1b, as is expected for samples with different surfaces exposed and small variations in measurement parameters between each experiment. However, the key quantity is the ratio of Se to Te, which we present in Supplementary Figure 1c. This reveals three important points: i) the Se/Te ratio is relatively consistent across different sample regions; ii) samples fabricated with 200 nm CdSe underlayers have significantly higher Se:Te ratios; and iii) within each sample the ratio of Se to Te is relatively uniform throughout the film thickness, with significant changes occurring only close to 200 etches i.e. extremely close to the glass substrate (see Supplementary Figure 1a/b). This shows that the vast majority of our films have relatively uniform proportions of Se and Te and that ratio can be controlled via the thickness of the initial CdSe layer. Finally, in Supplementary Figure 1c we present the approximate region of the sample which the photoluminescence originates from, following discussion in the main text.

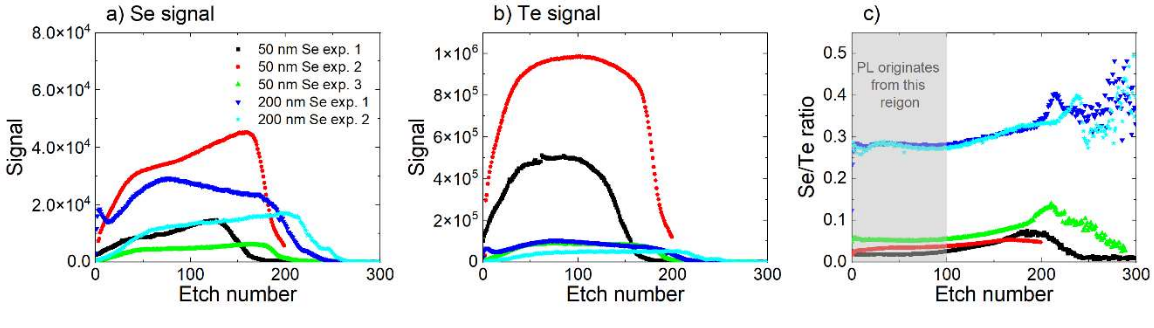


*Supplementary Figure 1. SIMS intensity for a) Se and b) Te as a function of Etch number (with 0 etches being the air-exposed surface). c) ratio of Se to Te intensities, indicating the region from which the photoluminescence (PL) originates. Legend in a) applies to all plots.*

## 2. *Interferometry thickness measurements*

To better understand our SIMS results, we carried out interferometry on the regions that we had etched. This allows us to estimate sample thickness. In all cases we applied a first order planefit to our data before calculating the thickness. We present results in Supplementary Table 1.

| CdSe underlayer thickness | Sample thickness (μm) | Standard deviation (μm) |
|---|---:|---:|
| 50nm experiment 1 | 2.69 | 0.11 |
| 50nm experiment 2 | 2.39 | 0.15 |
| 50nm experiment 3 | 2.40 | 0.12 |
| 200nm experiment 1 | 2.56 | 0.16 |
| 200nm experiment 2 | 2.65 | 0.16 |

*Supplementary Table 1. Sample thicknesses of regions etched in SIMS, as recorded through interferometry.*

## 3. *Atomic force microscopy*

We recorded Atomic Force Microscopy (AFM) maps of several regions of each sample to better understand their morphology, with representative maps presented in Supplementary Figure 2. We recorded the standard deviation in height for each AFM map, a measure of the surface roughness. We found that surface roughness was comparable in all samples, with results presented in Supplementary Table 2.

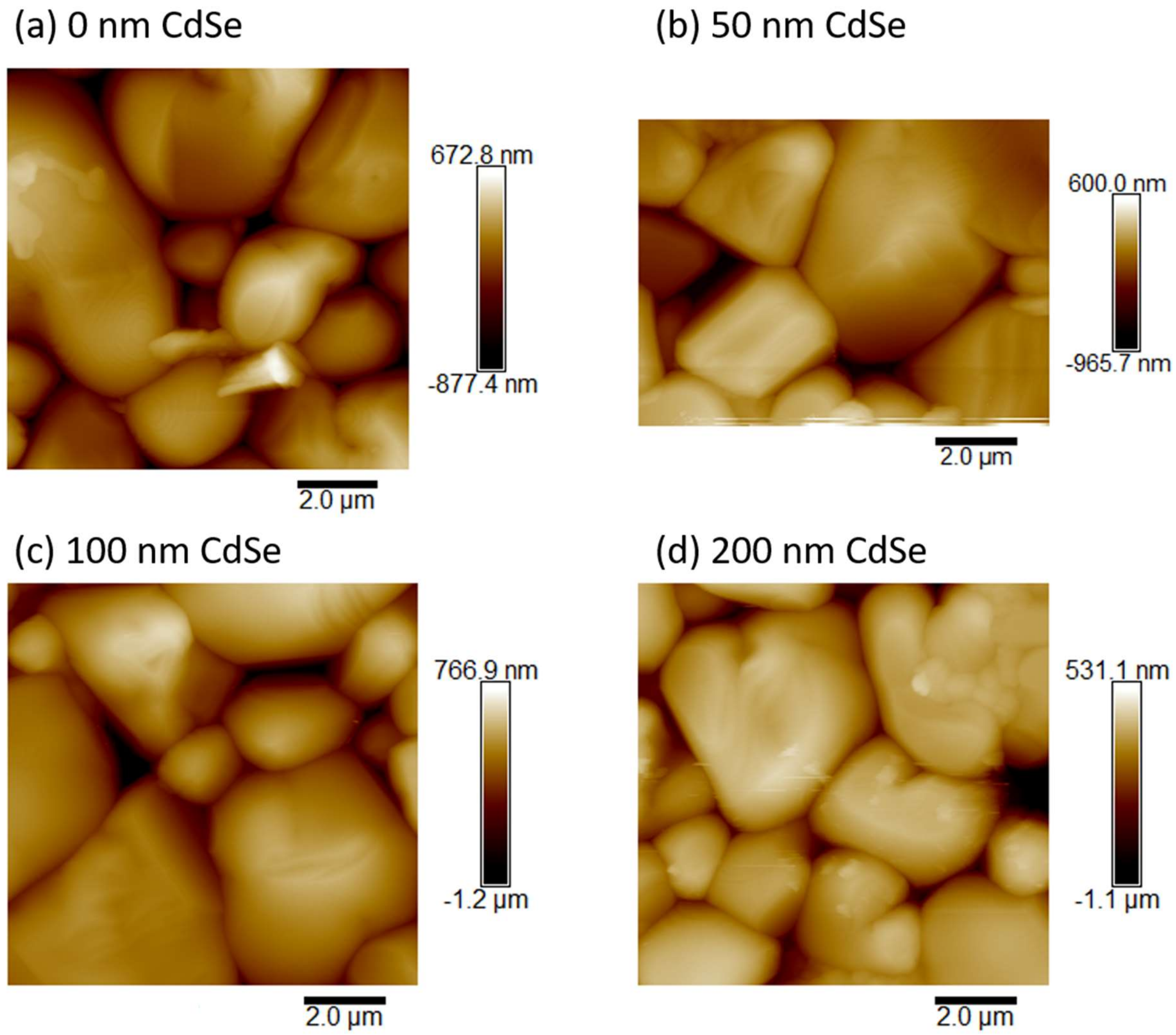


*Supplementary Figure 2. Representative AFM maps of Cl-treated 0 nm/50 nm/100 nm/200 nm CdSe samples.*

| *Sample (CdSe thickness, nm)* | *Average standard deviation in height, a measure of roughness, in AFM maps across samples measured (nm)* | *Standard deviation in roughness measurements (nm)* |
|---|---|---|
| *0* | *186* | *17* |
| *50* | *176* | *35* |
| *100* | *178* | *50* |
| *200* | *214* | *7* |

*Supplementary Table 2. AFM results for Cl-treated* $CdSe_xTe_{1-x}$ *samples.*

*Supplementary Information Note 3 – hyperspectral absorption measurements*

We also measured absorption across the sample using a hyperspectral imaging system[6] and obtained similar results to those presented in the main text.

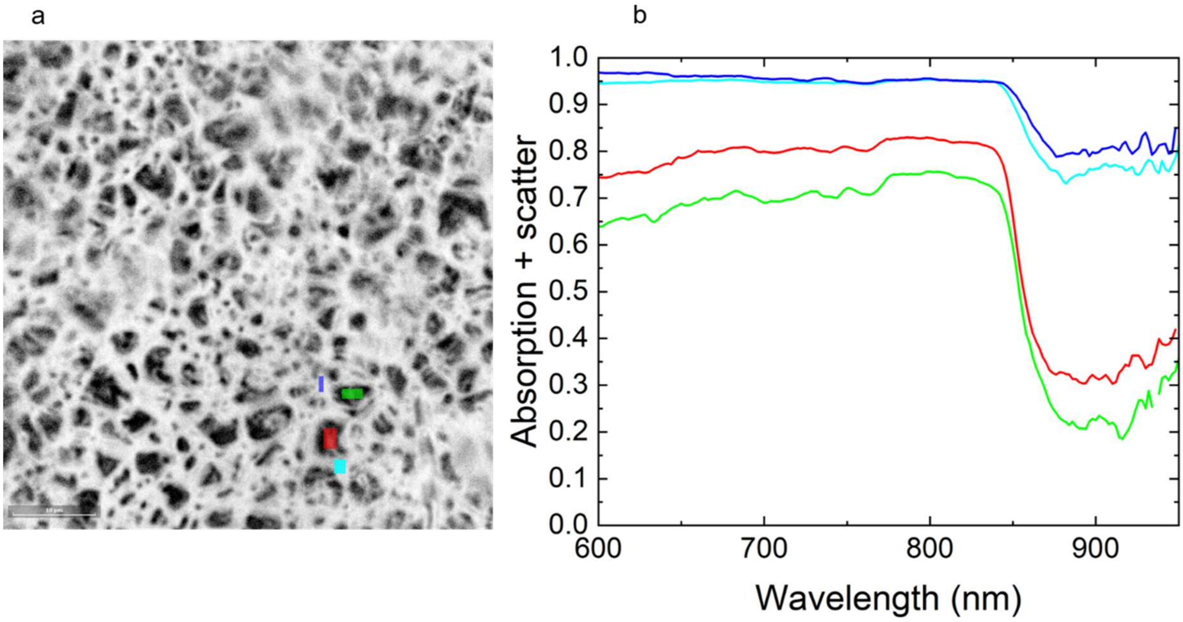


*Supplementary Figure 3. a) presents the absorption + scattering at the band edge for a Cl-treated 200 nm CdSe film. b) presents the absorption + scattering for the specific coloured regions highlighted in a). It can be seen that very similar results are observed to those in Figure 1 in the main text.*

*Supplementary Information Note 4 – average absorption measurements*

Supplementary Figure 4 presents the spatially averaged *absorption* + *scattering* for all samples. Supplementary Figure 5 presents Tauc fitting of below-bandgap scattering subtracted data, while Supplementary Figure 6 presents microscale *absorption* + *scattering* maps - the equivalent of Figure 1c - for other Cl-treated samples. It can be seen from these plots that, in the regions without grains present, the below-bandgap scattering is comparable for all samples.

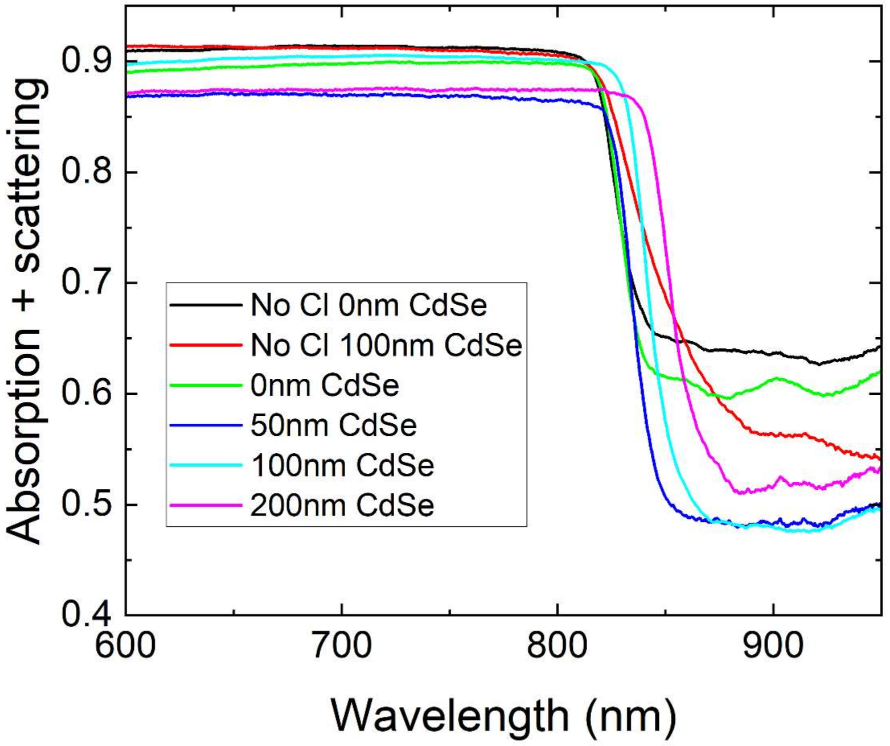


*Supplementary Figure 4. Average absorption for all samples for measurement series presented in the main text.*

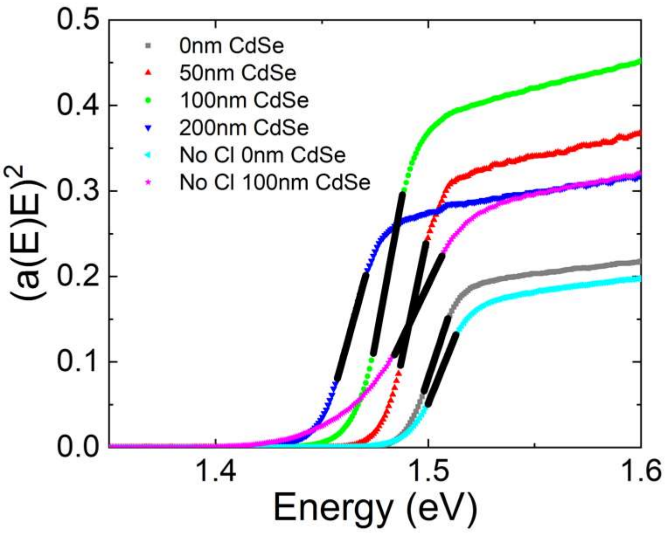


*Supplementary Figure 5. Tauc fits (black lines) to scattering subtracted absorption data.*

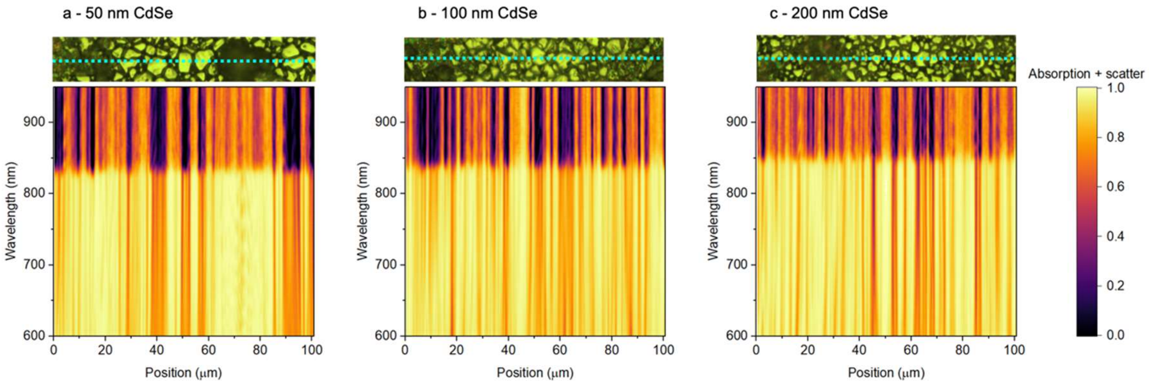


*Supplementary Figure 6. Absorption + scattering maps for Cl-treated 50 nm, 100 nm and 200 nm CdSe are presented in a), b) and c) respectively.*

*Supplementary Information Note 5 – Tauc fitting absorption measurements*

We carried out Tauc fitting of spatially resolved absorption measurements recorded for both lineslice measurements (Supplementary Figure 7) and the hyperspectral imaging system (Supplementary Figure 8). In the case of the lineslice, more variation in bandgap is observed for the (Cl-treated) 200 nm CdSe sample than the 0 nm CdSe sample, and decreases in bandgap roughly correlate with grain boundaries. However, subtraction of below-bandgap scattering prior to Tauc fitting introduces some noise in these measurements (as can be seen in Supplementary Figure 7, point to point variation is around 0.01 eV), preventing us from definitively stating that bandgaps are always lower at grain boundaries. In the hyperspectral approach we again observe more bandgap variation for 200 nm CdSe (see Supplementary Figure 8d compared to Supplementary Figure 8c). However, here the below-bandgap data was extremely noisy, especially between grains. Thus we were not able to carry out Tauc fitting at all regions of the map, again preventing us from drawing definitive conclusions. In both cases the data is strongly suggestive of lower bandgaps at grain boundaries.

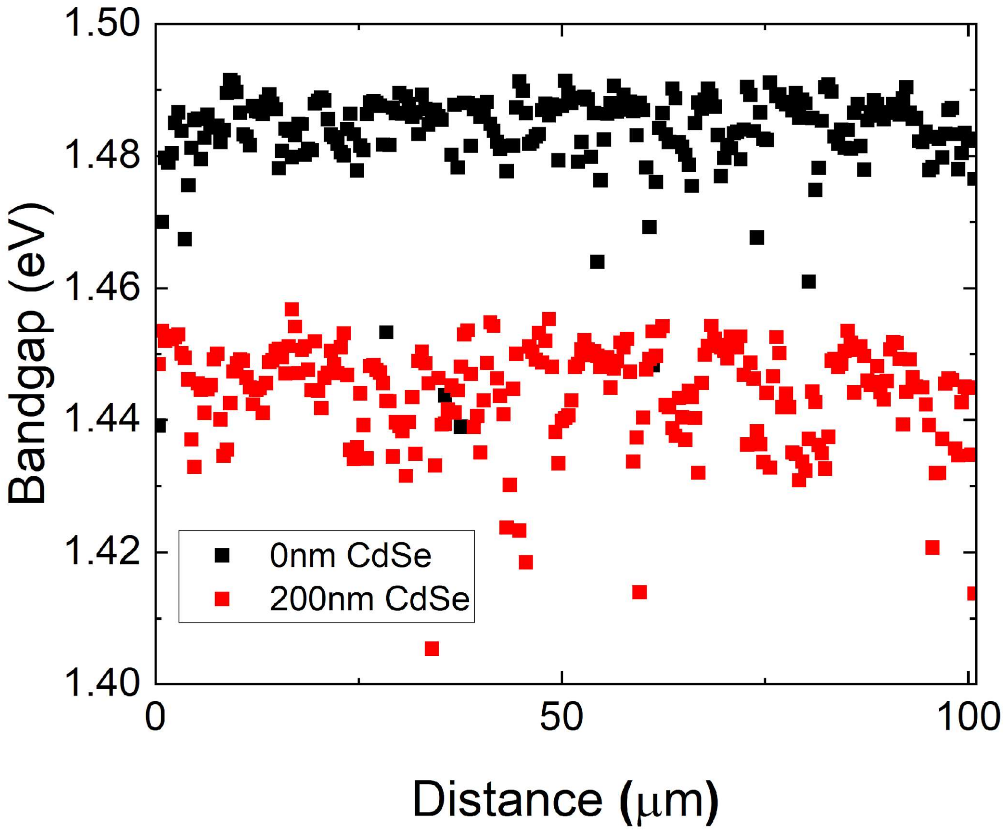


*Supplementary Figure 7. The fitted Tauc bandgap at all positions across the lineslice measured for Cl-treated 0 nm CdSe and 200 nm CdSe. It can be seen there is more bandgap variation for the 200 nm CdSe.*

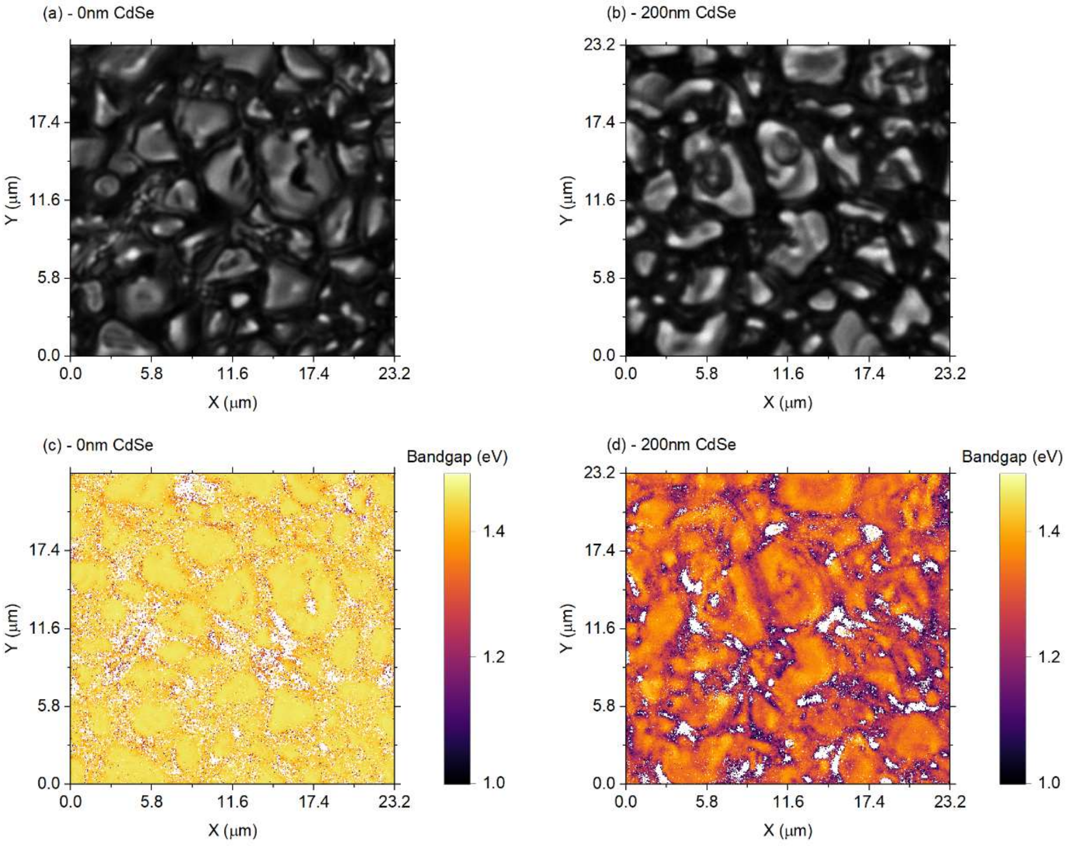


*Supplementary Figure 8. a)/b) reflection images for 600 nm illumination and c)/d) bandgaps extracted from Tauc fits of absorption plots for Cl-treated 0 nm/200 nm CdSe. White regions of c) and d) are where data was too noisy to confidently extract a bandgap.*

*Supplementary Information Note 6 – trap-dominated regime*

In steady state the local charge generation rate, $G$, is equal to the charge recombination rate $R$. Furthermore, for any position in the material $G \propto I$, the incident laser intensity, and $R \approx a(n - n_i) + b(n^2 - n_i^2) + c(n^3 - n_i^3)$, that is the recombination depends on first, second and third order recombination rates $a, b$ and $c$, where $n$ is the density of excited electrons and the subscript $i$ corresponds to intrinsic concentrations. We have assumed here that the density of excited electrons is approximately equal to the density of excited holes. Furthermore, we can state that (for an undoped system) the radiative recombination rate is $b_r(n^2 - n_i^2)$. Therefore, the recorded photoluminescence divided by the incident laser intensity is

$$\frac{PL_{recorded}}{I} \propto \frac{b_r(n^2 - n_i^2)}{a(n - n_i) + b(n^2 - n_i^2) + c(n^3 - n_i^3)} \approx \frac{b_r n}{a + bn + cn^2}$$

where for the last approximation we have assumed $n \gg n_i$, which is true for the excitation densities used in our experiments. Therefore, if we plot $\frac{PL_{recorded}}{I}$ versus $I$ and observe a positive gradient, this indicates we are operating in a trap-dominated regime where $R \approx an$ (as then $\frac{PL_{recorded}}{I} \propto I$). In Supplementary Figure 9a we plot the luminescence signal as a function of incident laser intensity, showing its shape does change with incident power. In Supplementary Figure 9b we plot $\frac{PL_{recorded}}{I}$ versus $I$ for excitation densities of the same order of magnitude as those used in our mapping experiments and we observe an approximate straight line, confirming that we are within a trap-dominated regime.

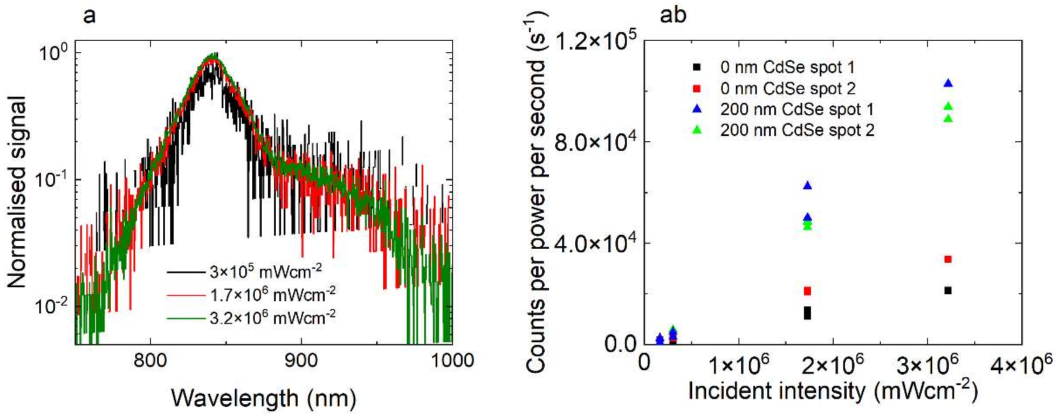


*Supplementary Figure 9. a) Normalised luminescence signal for a 200 nm CdSe sample as a function of the incident laser intensity. b)* $\frac{PL_{recorded}}{I}$ *versus I for different points on 0 nm CdSe and 200 nm CdSe samples, with Cl treatment. Linear scaling is observed in all cases. Power was increased from minimum to maximum and then reduced back to minimum to search for hysteresis effects, hence multiple points at the same intensity for some samples.*

*Supplementary Information Note 7 – prediction of photoluminescence from absorption measurements*

We applied the van Roosbroeck-Shockley relation[7] to predict the spectral shape of the photoluminescence from our absorption measurements. Specifically, this relation states that $PL(E) \propto a(E)E^2 e^{-\frac{E}{k_B T}}$, where $a(E)$ is the measured absorption as a function of energy $E$ and $k_B T$ the thermal energy, with the temperatures extracted in Supplementary Information Note 11 used. We used the scattering subtracted, spatially averaged $a(E)$ (i.e. that presented in Supplementary Figure 5) and found we had to shift predicted values up by 7 meV (i.e. predicted PL peaks were at lower energies than measured PL peaks) to obtain good agreement with spatially averaged measured photoluminescence. We attribute this shift to our PL measurements probing the front half of the $CdSe_xTe_{1-x}$ layer (see main text for discussion) while absorption measurements probe the entire $CdSe_xTe_{1-x}$ layer. As our SIMS results showed (Supplementary Information Note 2), near the glass substrate there is a small region with increased Se content, and thus marginally lower bandgap observed in absorption. Finally, we note that there is significant error in our below bandgap $a(E)$ measurements, so we only predict the energies close to the PL peak on the lower energy side. A comparison between experiment and theory is presented in Supplementary Figure 10, with the good agreement implying that there is minimal Stokes shift prior to photoluminescence.

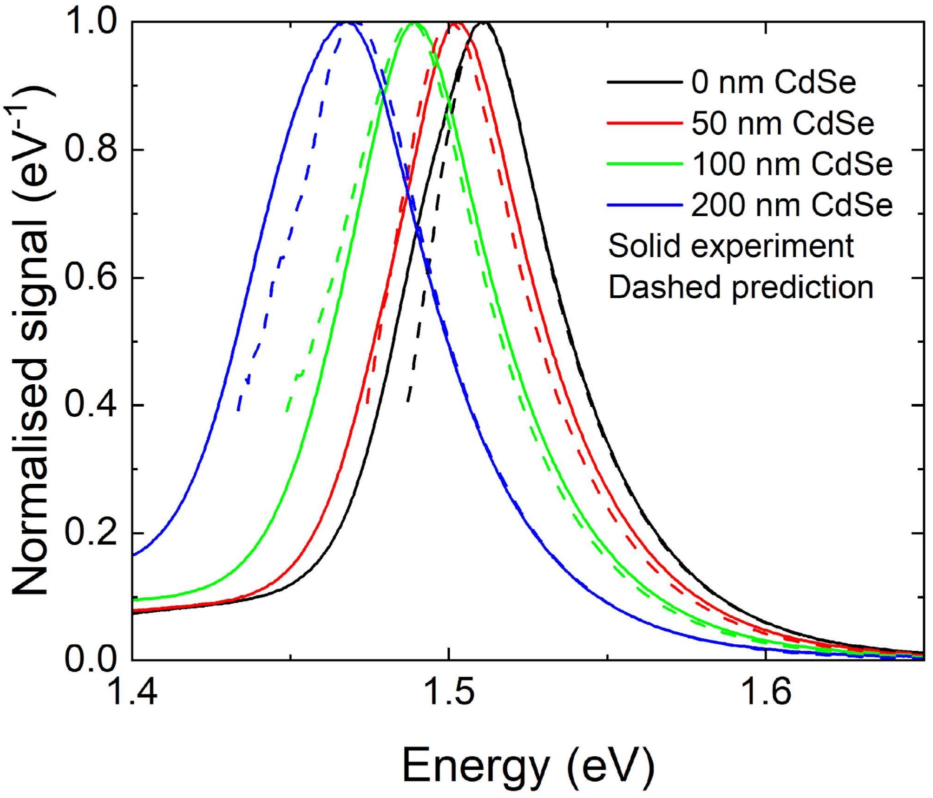


*Supplementary Figure 10. Comparison between measured (solid lines) and absorption predicted (dashed lines) photoluminescence for Cl-treated samples. Predicted PL was shifted by 7 meV to have good agreement (see text above).*

*Supplementary Information Note 8 – additional average PL information*

*i) Logarithmic scale spectra and Urbach fit*

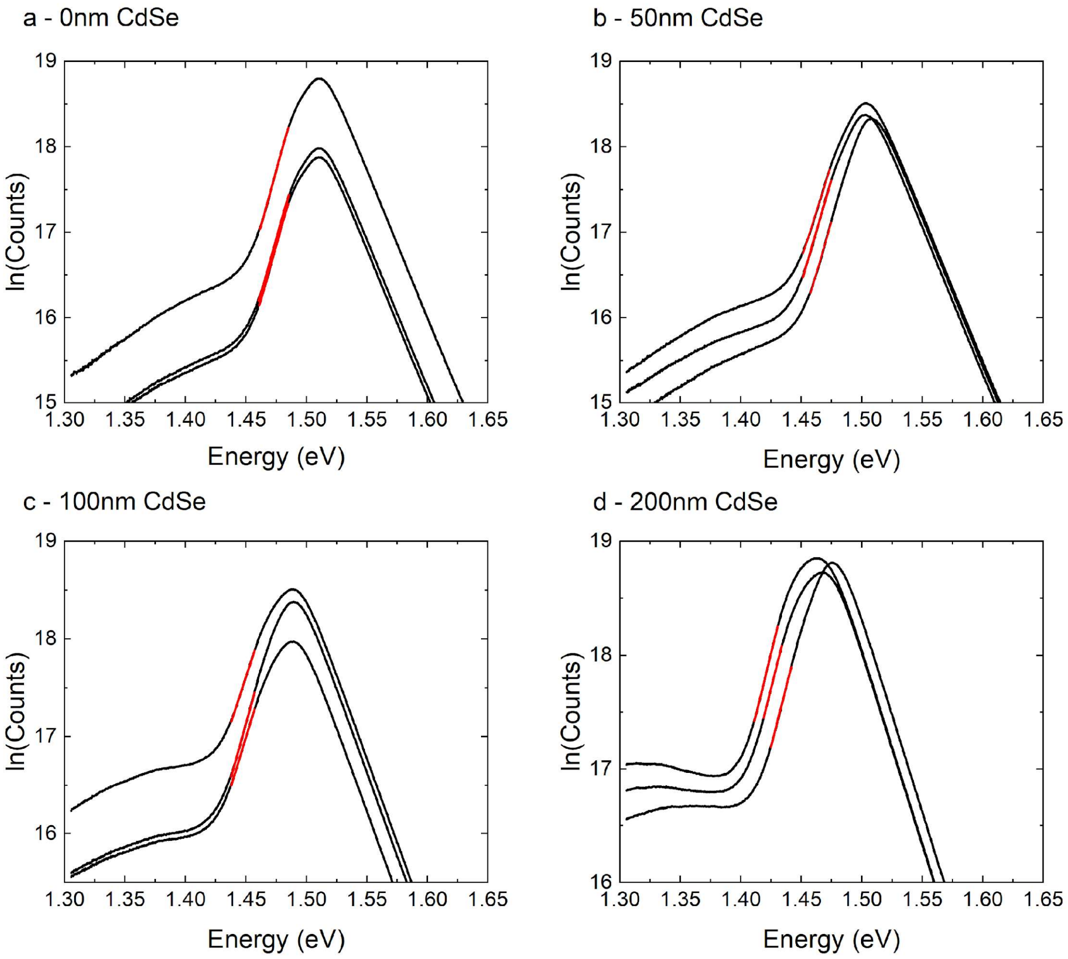


*Supplementary Figure 11. a)/b)/c)/d) the natural logarithm of the spatially averaged photoluminescence spectrum from three regions mapped for 0 nm/50 nm/100 nm/200 nm CdSe as a function of energy (black). Overlaid on each plot is the Urbach fit to the below bandgap region (red).*

*ii)   Uncalibrated data stretching to longer wavelengths*

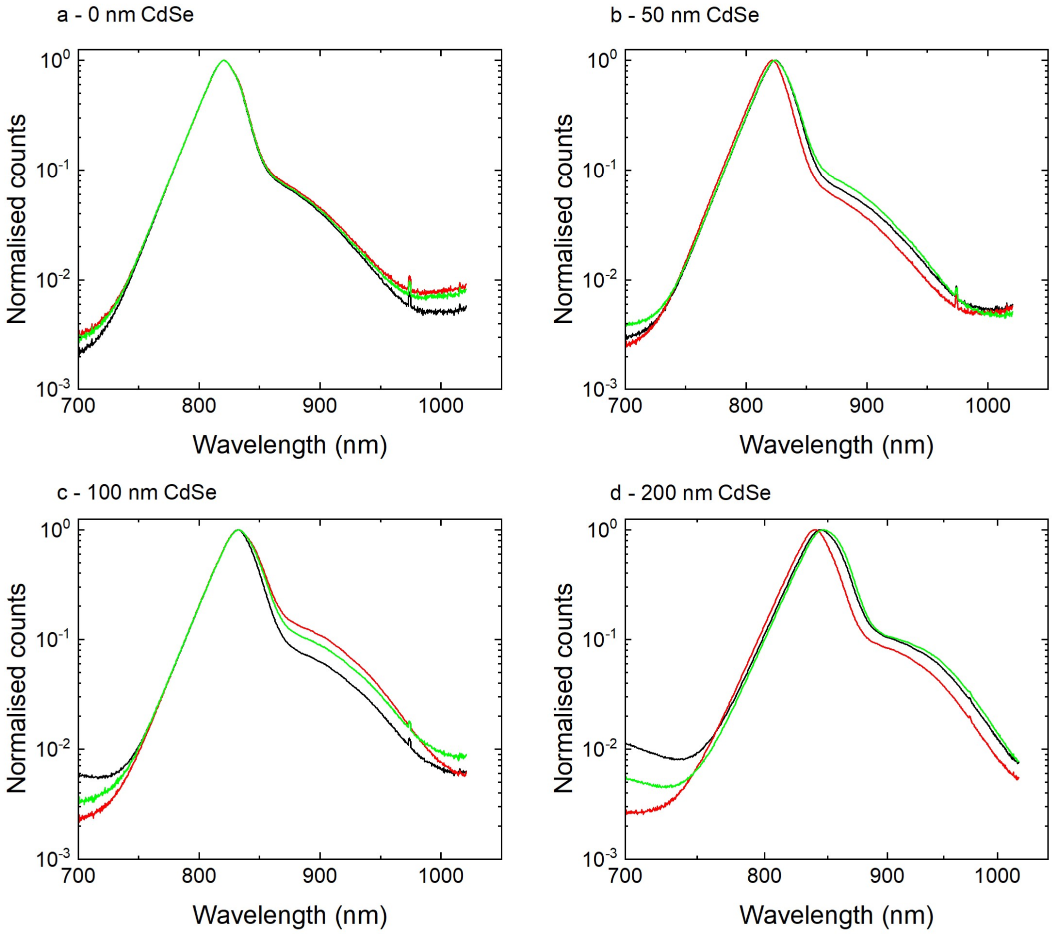


*Supplementary Figure 12. Uncalibrated average PL data for three measurements of each sample stretching to beyond 1000 nm, on a logarithmic scale. We note that we were confident in our absolute calibration only to 950 nm.*

*Supplementary Information Note 9 – other PL maps*

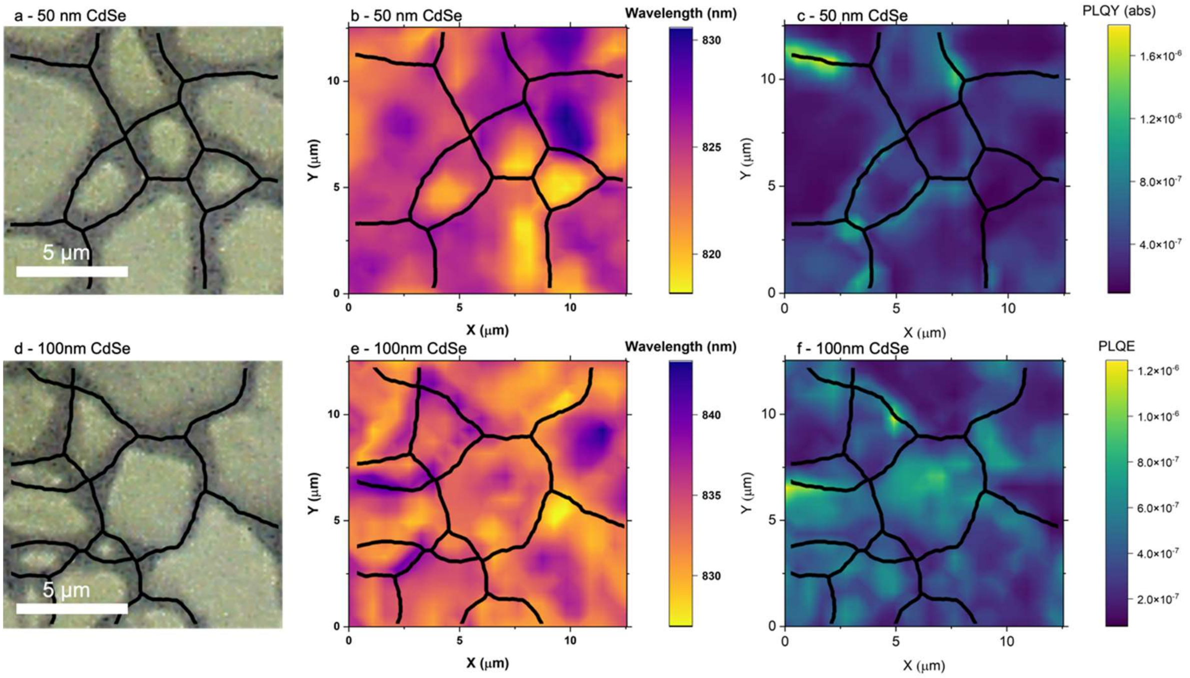


*Supplementary Figure 13. a), b) and c)/d), e) and f) present white light reflection, peak wavelength and peak PLQY plots (± 20 nm about the central peak) for Cl-treated 50 nm CdSe and 100 nm Cl-treated CdSe samples. We note that the same colour scales are used for different ranges in b)/e) and c)/f) to allow for better visualisation of results. As a guide to the eye, black lines present approximate grain boundaries.*

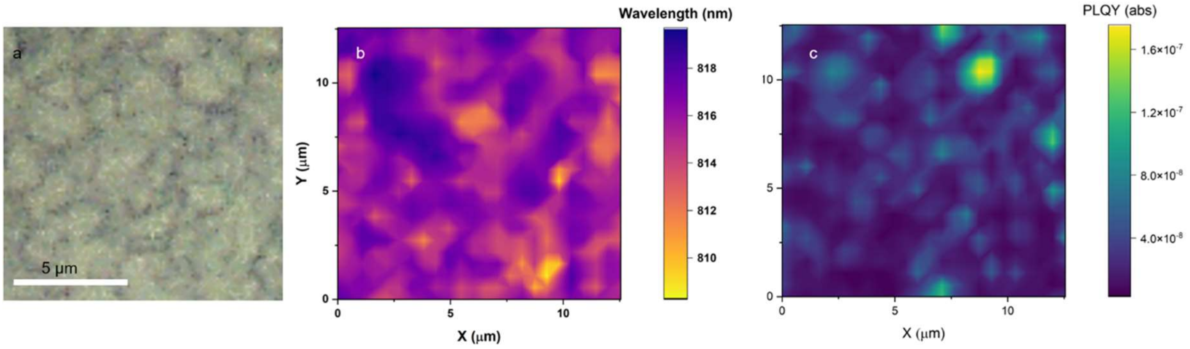


*Supplementary Figure 14. a), b) and c) present white light reflection, peak wavelength and peak PLQY plots (± 20 nm about the central peak) for untreated 0 nm CdSe.*

*Supplementary Information Note 10 – overlaid maps*

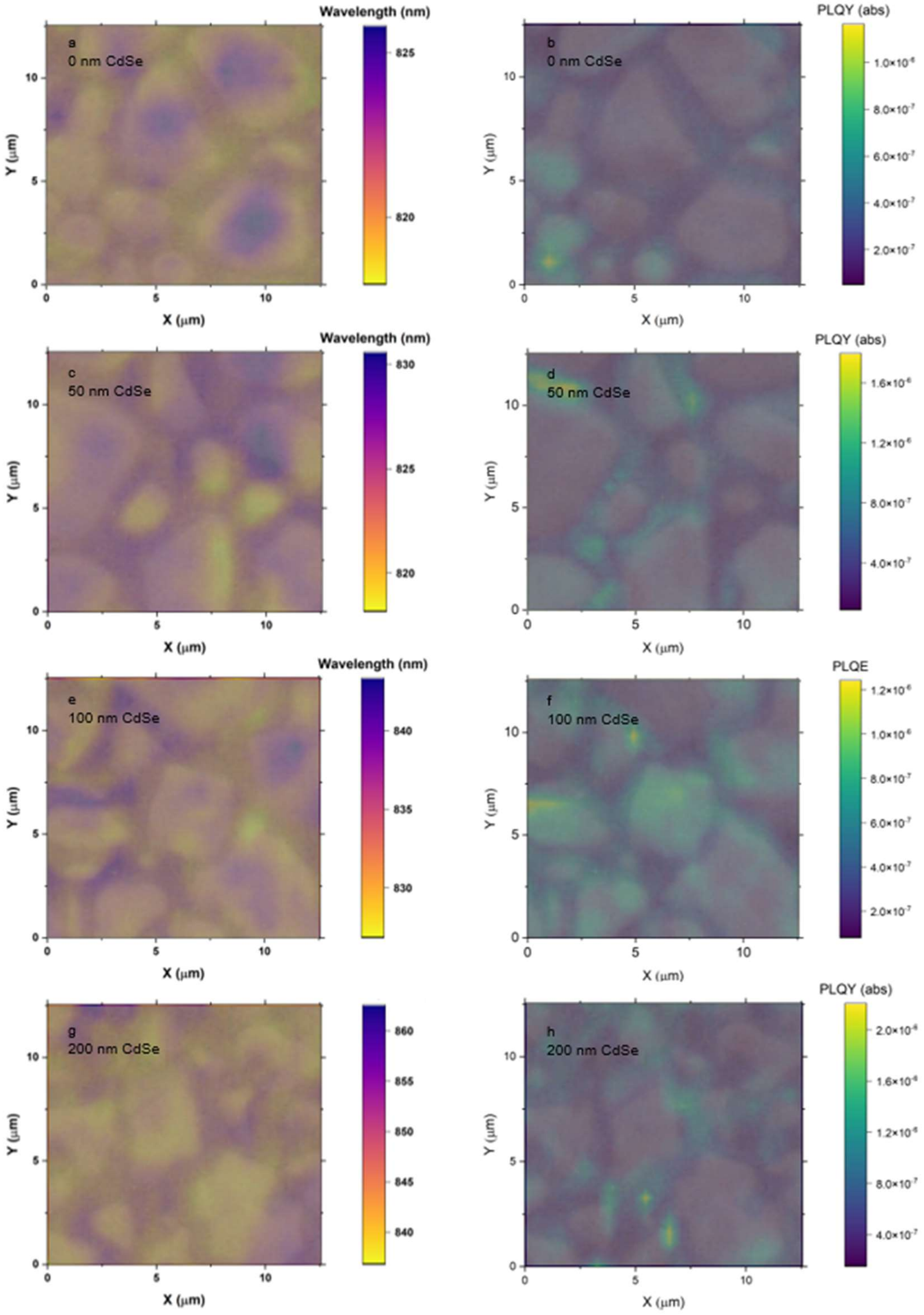


*Supplementary Figure 15. a)/c)/e)/g) and b)/d)/f)/h) present peak wavelength maps and PLQE maps overlaid with the white light sample reflection images for Cl-treated 0 nm/50 nm/100 nm/200 nm CdSe.*

*Supplementary Information Note 11 – further map analyses*

1. *Temperature fitting*

As $PL(E) \propto a(E)E^2 e^{-\frac{E}{k_B T}}$ (see Supplementary Information note 7), where $a(E)$ is the sample absorption, and $a(E)$ is approximately constant well above the bandgap, we can state that in this energy region $ln\left(PL(E)\right) = A - \frac{E}{k_B T}$, where $A$ is a constant. We fitted our PL maps to extract the local temperature for each sample, which we present in Supplementary Figure 16 for all samples. We find that all samples are at approximately 322 K during measurements except 200 nm CdSe, which is reasonable as we are using an intense laser beam to access the low PLQEs. We find 200 nm CdSe samples are approximately 4 K hotter, which we attribute to differences in the thermal diffusion coefficient with increased CdSe.

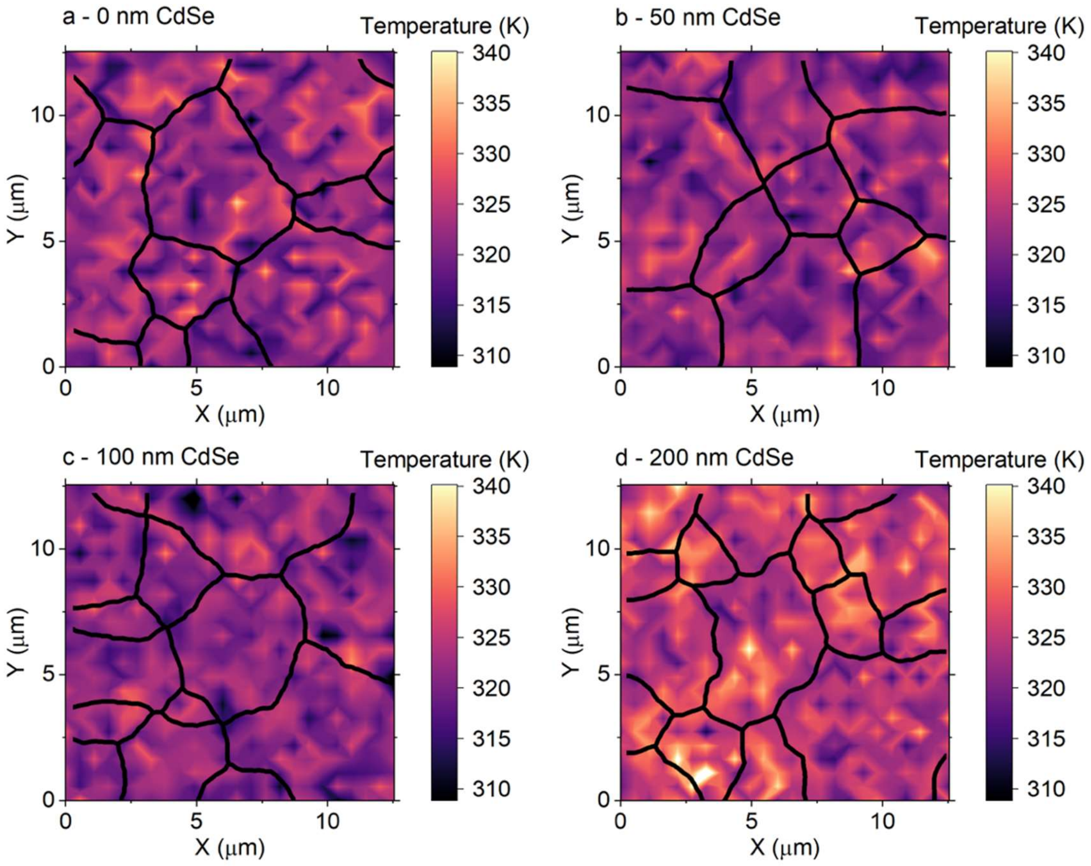


*Supplementary Figure 16. a)/b)/c)/d) Fitted temperature maps for 0 nm/50 nm/100 nm/200 nm CdSe underlayer.*

*2. Ideal radiative voltage*

Based on the data presented in Supplementary Figure 4 we can assume that $a(E)\sim0.9$ well above the bandgap. Furthermore, in an ideal system (i.e. with no sub-bandgap PL), we assume the absorption follows an Urbach fit below the bandgap. Using these assumptions, and noting $PL(E) \propto a(E)E^2e^{-\frac{E}{k_BT}}$, we extracted $a(E)$ from every point measured on our map (using the temperatures extracted above). We can therefore calculate the Shockley-Queisser radiative open circuit voltage noting

$$V_{OC,rad} = k_BT\ ln\ ln\left(\frac{J_{sc}}{J_r} + 1\right)$$

where $J_{sc} = q\int \phi_{AM\ .5}(E)a(E)dE$ and $J_r = q\pi\int \phi_{bb}(E)a(E)dE$, where $\phi_{AM\ .5}$ is the AM1.5 solar flux (per unit energy, per unit area) and $\phi_{bb}$ is the black body flux (per unit energy, per unit area, per unit solid angle)[8]. We present $V_{OC,rad}$ for our PL measurements in Supplementary Figure 17, for solar cells operating at 300 K. As expected, $V_{OC,rad}$ falls for samples with more Se present, and there is relatively small variation of $V_{OC,rad}$ across the surface of each sample, suggesting relatively uniform samples from an absorption perspective.

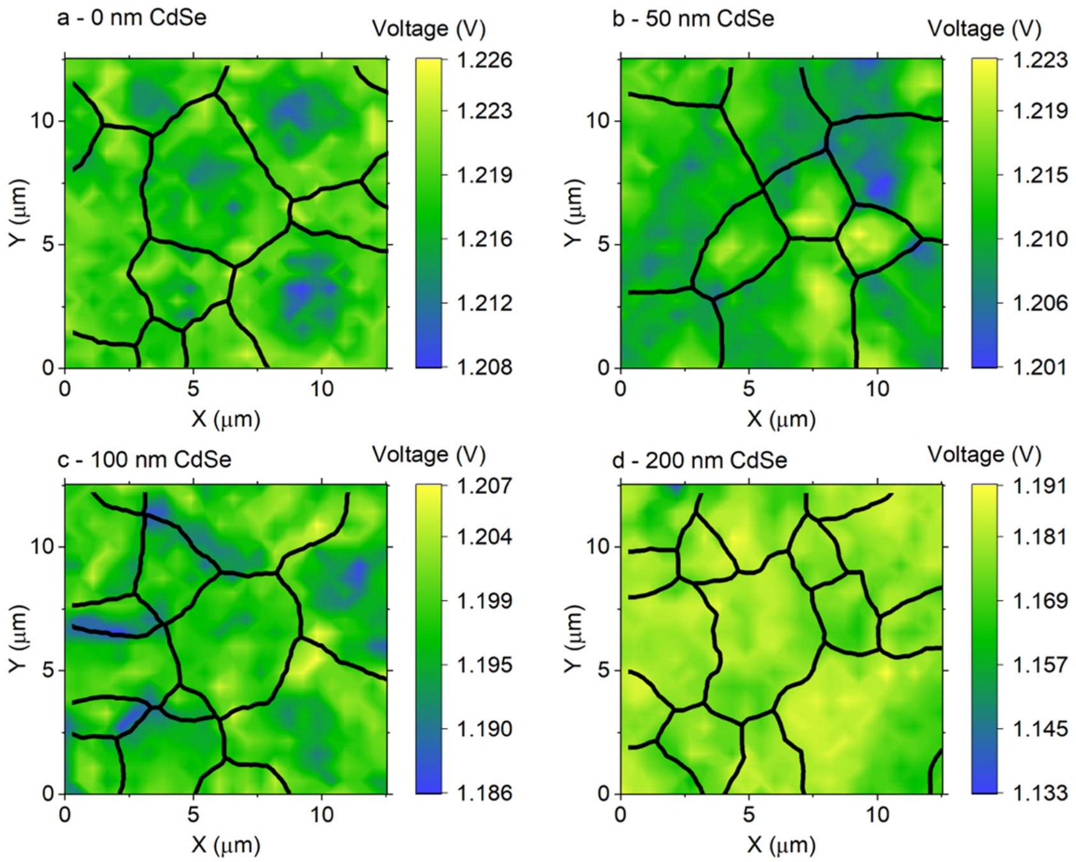


*Supplementary Figure 17. a)/b)/c)/d) Radiative limit open circuit voltage maps for 0 nm/50 nm/100 nm/200 nm CdSe underlayer. Note colour-bar is a different scale for each plot.*

*3. Implied open circuit voltage*

As noted in the main text, an implied open circuit voltage can also be calculated from measured PLQY values through $V_{OC} = V_{OC,ideal} + \frac{k_B T}{q} ln\,(PLQY)$ [8] Here we take the PLQY of the main luminescence peak (removing any effects of sub-bandgap luminescence) to calculate the maximum open-circuit-voltage achievable with measured films, which we present in Supplementary Figure 18, again for solar cells operating at 300 K. $V_{OC}$ is lower in grain interiors for 200 nm CdSe, despite the higher bandgaps present in these regions.

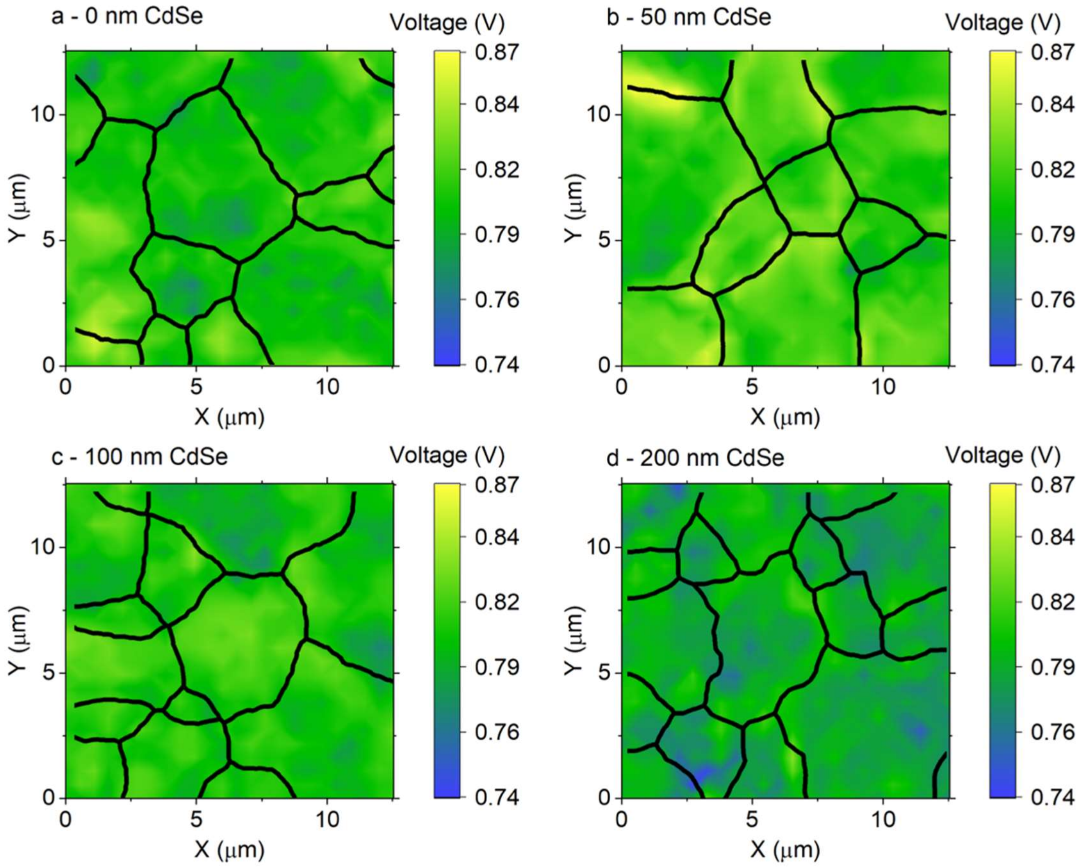


*Supplementary Figure 18. a)/b)/c)/d) Implied open circuit voltage maps for 0 nm/50 nm/100 nm/200 nm CdSe underlayer, neglecting any effect of the sub-bandgap defect luminescence.*

*4. Average values*

Here we present average values from the above analyses.

| CdSe thickness (nm) | Temperature (K) | $V_{OC,ideal}$ (V) | $V_{OC}$ (V) |
|---|---|---|---|
| 0 | 322 ± 1 | 1.2183 ± 0.0006 | 0.8070 ± 0.0032 |
| 50 | 322 ± 1 | 1.2121 ± 0.0004 | 0.8167 ± 0.0043 |
| 100 | 321 ± 1 | 1.1974 ± 0.0007 | 0.8062 ± 0.0025 |
| 200 | 326 ± 1 | 1.1772 ± 0.0018 | 0.7895 ± 0.0024 |

*Supplementary Table 3. Average results and standard deviation for fits in this supplemental note.*

*Supplementary Information Note 12 – correlation maps*

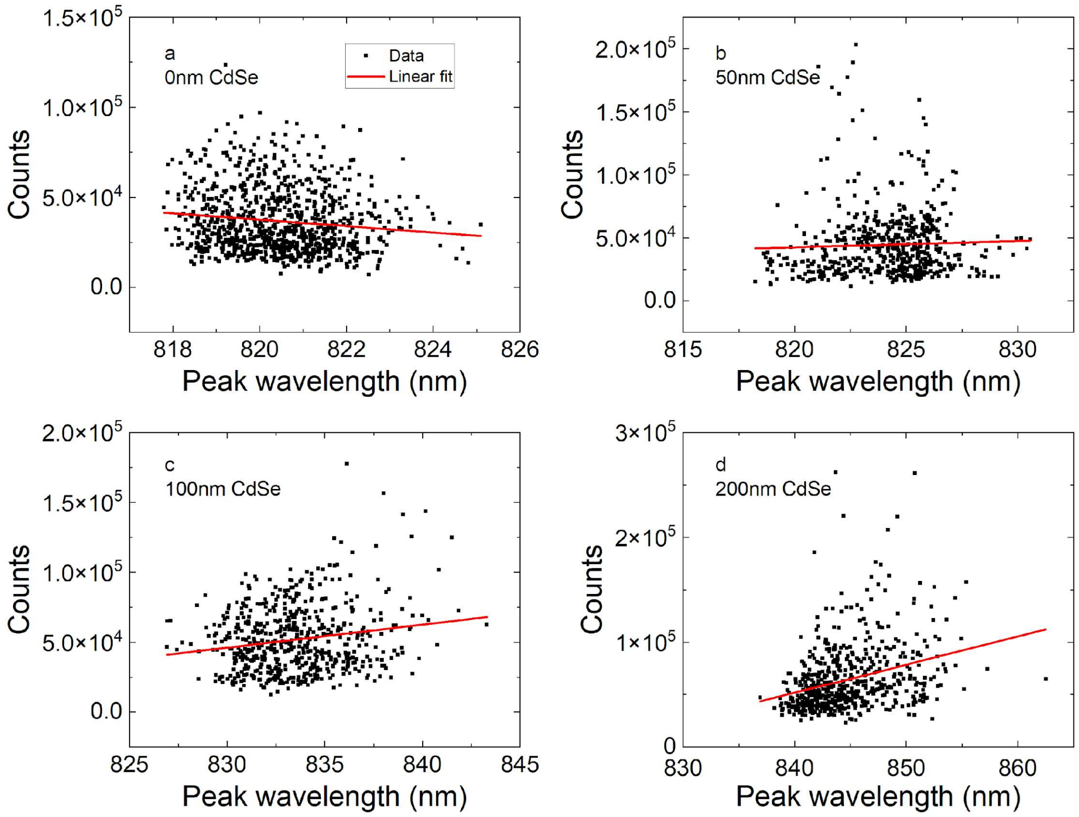


*Supplementary Figure 19. a)/b)/c)/d) present correlation maps between peak wavelength and total PL counts for Cl-treated 0 nm/50 nm/100 nm/200 nm CdSe.*

*Supplementary Information Note 13 – plots of long-wavelength PLQY*

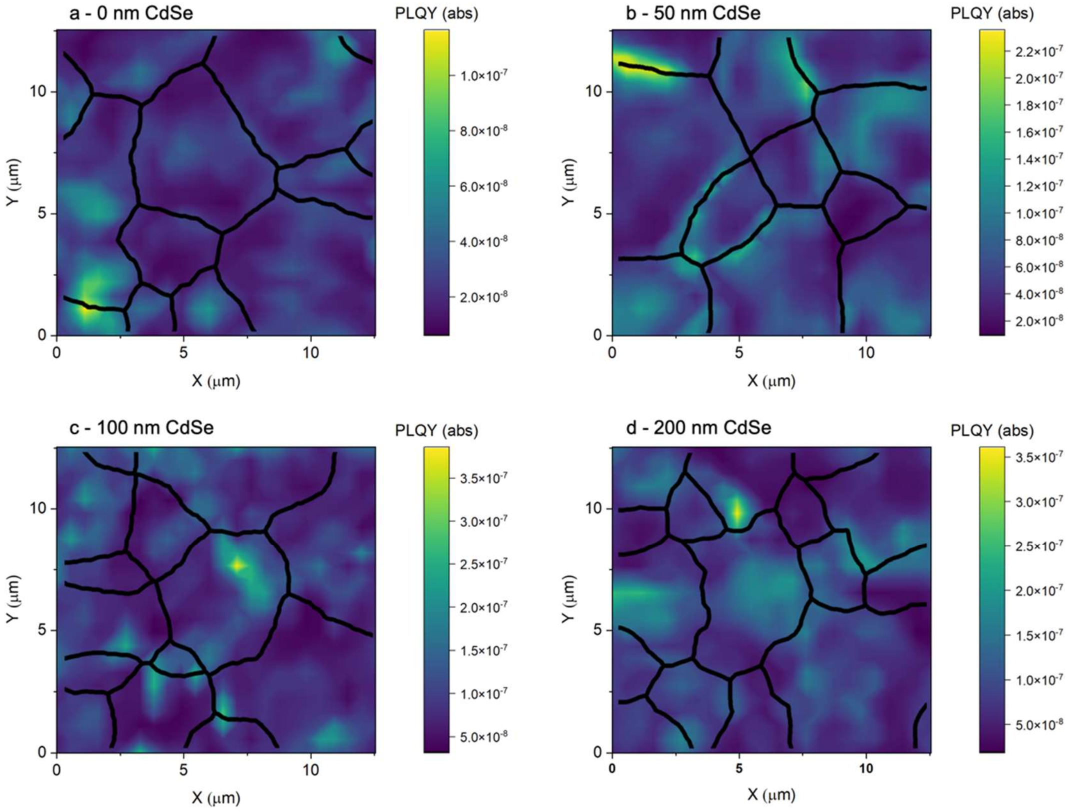


*Supplementary Figure 20. a)/b)/c)/d) presents long wavelength PLQYs for Cl-treated 0 nm/50 nm/100 nm/200 nm CdSe. PLQYs are for wavelength regions of 870 nm-950 nm/870 nm-950 nm/890 nm-950 nm/900 nm-950 nm respectively (chosen to avoid the edge of the main PL peak). As a guide to the eye, black lines present approximate grain boundaries.*

*References*


1. Stolterfoht, M. *et al.* Visualization and Suppression of Interfacial Recombination for High-Efficiency Large-Area Pin Perovskite Solar Cells. *Nat. Energy* **3**, 847 (2018).
2. Krückemeier, L., Liu, Z., Krogmeier, B., Rau, U. & Kirchartz, T. Consistent Interpretation of Electrical and Optical Transients in Halide Perovskite Layers and Solar Cells. *Adv. Energy Mater.* **11**, 2102290 (2021).
3. Krückemeier, L., Krogmeier, B., Liu, Z., Rau, U. & Kirchartz, T. Understanding Transient Photoluminescence in Halide Perovskite Layer Stacks and Solar Cells. *Adv. Energy Mater.* **11**, 2003489 (2021).
4. Ng, A. M. C., Djurišić, A. B., Chan, W.-K. & Nunzi, J.-M. Near Infrared Emission in Rubrene
5. Heterojunction Devices. *Chem. Phys. Lett.* **474**, 141 (2009).
6. Artegiani, E. *et al.* Effects of CdTe Selenization on the Electrical Properties of the Absorber for the Fabrication of CdSexTe1-x/CdTe Based Solar Cells. *Sol. Energy* **227**, 8 (2021).
7. Frohna, K. *et al.* Nanoscale Chemical Heterogeneity Dominates the Optoelectronic Response of Alloyed Perovskite Solar Cells. *Nat. Nanotechnol.* (2021).
8. van Roosbroeck, W. & Shockley, W. Photon-Radiative Recombination of Electrons and Holes in Germanium. *Phys. Rev.* **94**, 1558 (1954).
9. Ross, R. T. Some Thermodynamics of Photochemical Systems. *J. Chem. Phys.* **46**, 4590 (1967).
